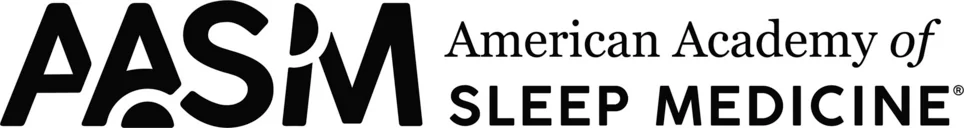# Selected abstracts from Sleep Medicine Disruptors 2025, a meeting of the American Academy of Sleep Medicine (AASM)

**DOI:** 10.1007/s44470-026-00114-7

**Published:** 2026-06-30

The American Academy of Sleep Medicine (AASM) held Sleep Medicine Disruptors 2025 at the Austin Marriott Downtown in Austin, Texas, and livestreamed the event Nov. 14 – 15, 2025. The chair of the meeting was Azizi Seixas, PhD, interim inaugural chair of the Department of Informatics and Health Data Science, founding director of The Media and Innovation Lab, associate director of the Center for Translational Sleep and Circadian Sciences, and director of population health informatics within the Institute for Data Science and Computing at the University of Miami Miller School of Medicine.

This biennial meeting, which was first held in 2019, attracts clinicians, scientists, technology developers, start-up founders, venture capitalists, and other healthcare innovators. Speakers explored technology innovation, artificial intelligence, and other disruptions that are poised to change the landscape of sleep health, patient care, and healthcare delivery. An exhibit hall also featured products and services from leading companies in the industry.

Robert Pearl, MD, former CEO of The Permanente Medical Group (Kaiser Permanente), presented the keynote address, “The Future of Healthcare,” on Friday, Nov. 14. He is the author of “ChatGPT, MD: How AI-Empowered Patients & Doctors Can Take Back Control of American Medicine.” On Saturday, Nov. 15, Natalie Nixon, PhD, presented the keynote, “From Uncertainty to Advantage: Harnessing Creativity to Navigate Ambiguity.” Other featured speakers were Feby Abraham, PhD; Allison Brager, PhD; Dennis Hwang, MD; David Kent, MD; Seema Khosla, MD; Charley Taylor, PhD; David White, MD; and Nathan Whitmore, PhD.

For the first time in 2025, the AASM issued a call for abstracts for Sleep Medicine Disruptors. Abstract submissions explored novel solutions, interventions, approaches, and paradigms related to topics such as sleep health monitoring, consumer sleep technology, sleep disorders screening, diagnosis and treatment, artificial intelligence and machine learning, healthcare delivery, and practice models. The 25 accepted abstracts were scheduled for poster presentation, and seven abstract authors were invited to give an in-person oral presentation.

The AASM also accepted submissions for the inaugural Sleep Medicine Disruptors Innovation Award, recognizing innovative products, features, and services that support sleep health and advance the practice of sleep medicine. The 23 entries in this new award program were evaluated by a panel of sleep medicine professionals, who selected eight finalists to give a pitch presentation during the meeting in Austin. In-person and livestream attendees voted to select the two winners: Bairitone Health’s anatomic polysomnogram won the diagnostics category, and Nidra by Noctrix Health was the winner of the therapeutics category.

**Sponsorship:** Publication of this supplement was funded by the American Academy of Sleep Medicine (AASM). All content was reviewed and approved by the Sleep Medicine Disruptors 2025 planning workgroup, which held full responsibility for the abstract selections.

**Program Listing**

*Friday, November 14, 2025*

8:00 AM – 8:15 AM CST Welcome & Overview by Azizi Seixas, PhD

8:15 AM – 9:00 AM CST Keynote Address: The Future of Healthcare by Robert Pearl, MD

9:00 AM – 9:30 AM CST Q&A with Azizi Seixas, PhD, and Robert Pearl, MD

9:30 AM – 10:00 AM CST Humans Over Hardware: Designing Sleep Tech that Works in the Real World by Allison Brager, PhD

10:00 AM – 10:15 AM CST Q&A with Azizi Seixas, PhD, and Allison Brager, PhD

10:15 AM – 11:15 AM CST Exhibits and Posters

11:15 AM – 12:15 PM CST Oral Abstract Presentations

12:15 PM – 1:30 PM CST Lunch Break

1:30 PM – 2:00 PM CST Bringing Computing and A.I. into Cardiology: From Bench to Business to Bedside by Charley Taylor, PhD

2:00 PM – 2:15 PM CST Q&A with Azizi Seixas, PhD, and Charley Taylor, PhD

2:15 PM – 2:45 PM CST From Rules-Based Tools to AI Agents: The Evolution of AI in Sleep Medicine by Dennis Hwang, MD

2:45 PM – 3:00 PM CST Q&A with Azizi Seixas, PhD, and Dennis Hwang,  MD

3:00 PM – 3:30 PM CST Break and Exhibits

3:30 PM - 4:00 PM CST The Next Generations of Consumer Sleep Technology: From Passive Monitoring to Autonomous Intervention by Nathan Whitmore, PhD

4:00 PM - 4:15 PM CST Q&A with Azizi Seixas, PhD, and Nathan Whitmore, PhD

4:15 PM - 5:15 PM CST Sleep Medicine Disruptors Innovation Award Contest Pitch Presentations

5:15 PM - 5:30 PM CST Innovation Award Contest Voting

5:30 PM - 6:30 PM CST Innovation Awards Reception

*Saturday, November 15, 2025*

8:00 AM – 8:15 AM CST Welcome and Recap by Azizi Seixas, PhD

8:15 AM – 9:00 AM CST Keynote Address: From Uncertainty to Advantage — Harnessing Creativity to Navigate Ambiguity by Natalie Nixon, PhD

9:00 AM – 9:30 AM CST Q&A with Azizi Seixas, PhD, and Natalie Nixon, PhD

9:30 AM – 10:00 AM CST How Health Systems Can Transform for the Future by Feby Abraham, PhD

10:00 AM – 10:15 AM CST Q&A with Azizi Seixas, PhD, and Feby Abraham, PhD

10:15 AM – 11:15 AM CST Exhibits and Posters

11:15 AM – 12:15 PM CST Oral Abstract Presentations

12:15 PM – 1:30 PM CST Lunch Break

1:30 PM – 2:00 PM CST Pharma Therapy of OSA: Drug Targets and Current Outcomes Data by David White, MD

2:00 PM – 2:15 PM CST Q&A with Azizi Seixas, PhD, and David White, MD

2:15 PM – 2:45 PM CST Beyond Hypoglossal Nerve Stimulation: Future Directions for Neuromodulation in Obstructive Sleep Apnea by David Kent, MD

2:45 PM – 3:00 PM CST Q&A with Azizi Seixas, PhD, and David Kent, MD

3:00 PM – 3:30 PM CST Break and Exhibits

3:30 PM - 4:30 PM CST Sleep Medicine Disruptors Innovation Award Contest Pitch Presentations

4:30 PM - 4:45 PM CST Innovation Award Contest Voting

4:45 PM - 5:30 PM CST Talking Sleep Live with Seema Khosla, MD

5:30 PM - 5:45 PM CST Innovation Award Presentation & Closing with Azizi Seixas, PhD

**Oral Presentations**

**001 — Digital cognitive-behavioral therapy for insomnia: positive results maintained at 18- and 24-months after treatment**

Presenter: Frances P. Thorndike, Nox Research

Frances P. Thorndike^1^, Charles M. Morin^2^, Joseph M. Ojile^3^, Lee M. Ritterband^4^, Robert W. Gerwien^5^, Jason C. Ong^1^, Aleena M. Sange^6^, Samantha M. Edington^1^, Emerson M. Wickwire^7^, Heidi D. Riney^1^

^1^Nox Research, Nox Health, Alpharetta, GA; ^2^Laval University, Quebec, Canada; ^3^Clayton Sleep Institute, St. Louis, MO; ^4^University of Virginia School of Medicine, Charlottesville, VA; ^5^Independent Biostatistician, Newington, CT; ^6^Georgia Tech University, Atlanta, GA; ^7^University of Maryland School of Medicine, Baltimore, MD

**Introduction:** Although insomnia is one of the most prevalent sleep disorders (10-15% of adults), few receive first-line therapy (Cognitive-Behavioral-Therapy for Insomnia, CBT-I). CBT-I is an effective insomnia treatment and can improve co-occurring chronic conditions. Unfortunately, barriers prevent broad access to CBT-I. FDA-cleared digital therapeutics represent one innovative approach to overcoming limitations of traditional care models and increasing access to care. Even so, the durability of long-term effectiveness of digital CBT-I (dCBT-I) in real-world settings remains unclear. This study (NCT04325464) evaluated the effectiveness of Somryst, an FDA-cleared dCBT-I for chronic insomnia, over 18- and 24-months.

**Methods:** 1752 adults with chronic insomnia met eligibility in this single-arm study. Participants averaged 48.2 (*sd*=13.9) years; 77.1% were female. Insomnia severity was assessed using the Insomnia Severity Index (ISI) at baseline, end of treatment (EOT), and 6, 12, 18, and 24 months post-treatment. Depression and anxiety symptoms were measured at each assessment using established measures (PHQ-8, GAD-7). Longitudinal changes were analyzed using MMRM with time as a fixed effect and subjects as a random intercept.

**Results:** ISI scores significantly improved from a baseline mean of 19.1 (*sd=*4.03) to an EOT mean of 11.5 (*sd=*6.17) (LSM reduction: –7.21, p<0.001), with sustained effects at 24 months (-5.95, p<0.001). All post-assessment time points showed clinically significant ISI improvements compared to baseline (ps<0.001). At 24 months, 45.6% of participants were considered treatment responders (ISI change score ≥7). Improvements in depressive and anxiety symptoms were also observed at EOT (p<0.001), with sustained effects at 24 months (p<0.001).

**Conclusion:** In this real-world study**,** dCBT-I (Somryst) was associated with sustained improvements in insomnia severity, depressive symptoms, and anxiety symptoms to 24 months post-treatment. These findings are consistent with RCTs of dCBT-I and demonstrate durability in real-world settings.

While funding prohibited the full cohort from reaching the 2-year assessment, the study was well-powered to detect treatment effects at 24-months. Future research should examine how sleep improvements relate to additional health outcomes and potential changes in healthcare utilization and economic impact.

**Support:** Study was initially funded by Pear Therapeutics and later funded by Nox Health, which supported analysis and writing of this abstract.

**002 — Scaling CBTI with a clinical decision support platform: findings from a non-inferiority trial comparing CBTI to asynchronous, provider-guided CBTI**

Presenter: Anne Germain, NOCTEM Health

Anne Germain^1^, Megan Wolfson^1^, Brian O’Reilly^2^, Matthew S. Brock^3^, Hunter Hearn^4^, Vincent Mysliwiec^5^

^1^NOCTEM Health, Pittsburgh, PA; ^2^Madigan Army Medical Center, Tacoma, WA; ^3^USAF, Wilford Hall Ambulatory Surgical Center, San Antonnio, TX; ^4^NF/SG Veterans Affairs Medical Center, Gainesville, FL; ^5^Department of Psychiatry and Behavioral Sciences, The University of Texas Health Science Center at San Antonio, San Antonio, TX

**Introduction:** While cognitive-behavioral therapy (CBTI) is the first-line therapy for insomnia, its scalability is limited, which also limits patients’ timely access to care. Leveraging a clinical decision support (CDS) platform designed to facilitate provider-supervised, remote, asynchronous, and personalized CBTI delivery may increase the scalability, reach, and accessibility of CBTI. This non-inferiority trial compared the benefits and acceptance of CBTI as usual (CAU) and provider-supervised, asynchronous CBTI (A-CBTI) delivered via a CDS platform.

**Methods:** After completing a full sleep evaluation, patients who received a diagnosis of insomnia were randomized to CAU (n = 38) or A-CBTI (n = 38). CBTI was delivered by doctoral-level clinical psychologists in both groups. The CAU group received weekly in-person CBTI, whereas A-CBTI was delivered weekly via a clinical decision support platform (COAST®, NOCTEM® Health, Inc. 2022). The primary outcome was the changes in insomnia severity from baseline (T1) to 6 weeks (T2) and 3 months (T3). The non-inferiority margin (NIM) for the group difference in changes in insomnia scores over time was set a *d* = 0.5 (*p* = 0.025). Patients’ ratings of their experience in treatment were also compared.

**Results**: All 38 A-CBTI participants (10 women; mean age = 32.9 + 8.5; 20 endorsing current shift work) and all 38 CAU patients (8 women; mean age 31.3+ 7.3; 13 endorsing current shift work) initiated treatment. Follow-up data was available for 36 CAU participants and 34 A-CBTI participants. Both groups showed significant reductions in insomnia over time. The upper bound of the confidence intervals for the changes from T1 to T2 (d = -0.5; 95% CI [-0.908, -0.102) and T1 to T3 (d= -0.50 (-0.903, -0.098) was both below the NIM and 0, indicating that A-CBTI was both non-inferior and superior to CAU. Patients’ experience did not differ between groups.

**Conclusion:** A-CBTI delivered via a clinical decision support platform is non-inferior to traditional CBTI. A-CBTI offers a novel approach to address the CBTI capability gaps and offers an additional option in a stepped care model of insomnia.

**Support:** CDMRP Award # W81XWH-22-2-0008, JWMRP Log# JW210372, PI: Germain

**003 — Clinically integrated value-based care promotes efficient access to sleep treatment**

Presenter: Jason Ong, Nox Research, Nox Health

Jason C. Ong^1^, Rebecca Hankla^1^, Alp Sinan Baran^1^, Karina Hauser^1^, Jon Agustsson^2^ , Samantha Edington^1^, Frances Thorndike^1^, Heidi Riney^1^

^1^Nox Research, Nox Health, Atlanta, GA; ^2^Nox Research, Nox Medical ehf, Reykjavík, Iceland

**Introduction:** Value-based care is a paradigm-disrupting healthcare delivery model emphasizing patient outcomes and cost-effectiveness with the potential to overcome typically siloed management of chronic sleep disorders. We developed a virtual sleep healthcare model to evaluate and treat a range of sleep disorders including obstructive sleep apnea and insomnia. This study assessed the accessibility of this model for rural patients, who are often disadvantaged by traditional models of care.

**Methods:** Data were collected from patients presenting for screening between 2021 and 2023 to a telehealth, value-based sleep service. Patients diagnosed with chronic insomnia were prescribed digital CBT-for-insomnia when indicated. Patients suspected of obstructive sleep apnea (OSA) were first ordered a home sleep apnea test (HSAT). Those who tested positive for OSA were subsequently offered positive airway pressure (PAP) therapy with telehealth monitoring, supported by a clinically integrated care team composed of a board-certified sleep physician, nurse practitioner, respiratory therapist, and behavioral care manager. The time spent in successive sleep treatment steps was evaluated for both rural and urban patients.

**Results:** Of 42,687 patients, two-thirds (67.0%) lived in rural zip codes. Median length of days showed that rural patients typically underwent consultation with a board-certified physician within 6 days of screening, with urban patients seeing a physician within 5 days. Rural and urban patients completed HSAT within 12 days from physician consultation (ordered, mailed, completed, scored). Median values showed that sleep testing results were reviewed within 8 days of completing HSAT for rural patients (and 9 days for urban patients). Rural patients typically initiated PAP therapy within 6 days of receiving their diagnosis and recommended treatment plan (8 days for urban patients).

**Conclusion:** Real-world data of a comprehensive, clinically-integrated care model suggests rural patients gain access to quality sleep care at a rapid and comparable speed to urban patients. These findings suggest that this virtual sleep healthcare model can improve access and speed of care for patients irrespective of geography with potential economic benefits.

**Support:** This study was funded by Nox Health.

**004 — Improving diagnostic accuracy in home sleep apnea testing with Nox AI scoring (DeepRESP K241960)**

Presenter: Jason Ong, Nox Research, Nox Health

Jon S. Agustsson^1^, Ernir Erlingsson^1^, Guðný Árnadóttir^1^, Daníel Wilcox^1^, Kristófer Montazeri^1^, Eydís Arnardóttir^1^, Sigurður Ægir Jónsson^1^, Eysteinn Finnsson^1^, Þóra Björg Sigmarsdóttir^1^, Vaka Valsdóttir^1^, Rósa Hugosdóttir^1^, Megan McPhee^1^, Enisa Dresevic^1^, Jason Ong^2^, Sam Edington^2^, Frances Thorndike^2^, Heidi Riney^2^

^1^Nox Research, Nox Medical ehf, Reykjavík, Iceland; ^2^Nox Research, Nox Health, Atlanta, GA

**Introduction:** Home sleep apnea testing (HSAT) has expanded access but can exacerbate disparities: patients whose hypopneas end in arousals (rather than desaturations), those with comorbidities, and individuals for whom oximetry is less reliable (e.g., due to skin pigmentation or medications) are more likely to receive inconclusive results and delayed treatment. Nox AI Scoring (DeepRESP K241960), an FDA-cleared AI device, augments HSAT by adding automated sleep staging and arousal scoring, aligning HSAT outputs more closely with PSG.

**Methods:** We evaluated n=3,488 patient sleep studies from an AASM-accredited center, comparing automated HSAT-style rescoring against expert-scored PSG. Nox AI Scoring produced HSAT metrics with automated sleep staging and arousal scoring; the predicate device produced HSAT metrics without sleep staging/arousal scoring. We computed sensitivity, specificity, and accuracy versus PSG at AHI thresholds of 5, 15, and 30 events/hour, and quantified performance gains attributable to sleep staging/arousal scoring. The dataset represents the largest validation set used for FDA clearance of a sleep diagnostic device.

**Results:** Across all thresholds, Nox AI Scoring demonstrated high performance: at AHI ≥5, sensitivity 93.1%, specificity 81.1%, accuracy 92.5%; at AHI ≥15, sensitivity 82.1%, specificity 92.3%, accuracy 84.7%; at AHI ≥30, sensitivity 75.9%, specificity 96.8%, accuracy 87.2%. Incorporating sleep staging/arousal scoring improved sleep apnea detection from HSAT substantially: +10.7% sensitivity at AHI ≥5, +21.6% at AHI ≥15, +26.5% at AHI ≥30, with accuracy gains up to +16.8%. These improvements reduced false negatives and enabled HSAT outcomes to be deemed conclusive, rather than “negative” below threshold, thereby decreasing the need for additional testing prior to diagnosis.

**Conclusion:** Nox AI Scoring (DeepRESP K241960) materially increases HSAT sensitivity, specificity, and accuracy, transforming many traditionally inconclusive HSAT results into conclusive diagnostic outcomes and helping mitigate existing disparities in access and accuracy of sleep apnea diagnosis.

**Support:** This study was supported by Nox Health.

**005 — PromptNLP: electronic health record phenotyping using regular expressions derived from large language models**

Presenter: Nathanael Hwang, Harvard Medical School, Kaiser Permanente Southern California, Scaled Insights

Nathanael Hwang^1,2,3^, Haoqi Sun, PhD^1^, Robert Thomas^1^, Kendra A. Becker^2^, Dennis Hwang^2^, Matt Gratton^4^, Jessica Jara^2^, Joseph B. Kim^2^, Matthew T. Klimper^2^, Diego Mazzotti^4^, Anupamjeet Sekhon^2^, Jiaxiao Shi^2^, Rui Yan^2^, Olivia Hwang^3^, M. Brandon Westover^1^

¹Harvard Medical School, Boston, MA; ²Kaiser Permanente Southern California, Fontana, CA; ^3^Scaled Insights, Phoenix, AZ; ^4^University of Kansas Medical Center, Kansas City, KS

**Introduction:** Epidemiologic and machine learning research relies on structured data. Approximately 80% of relevant patient information remains in unstructured formats (i.e. clinical notes and test reports). Natural language processing (NLP) techniques, such as regular expression (regex), are labor-intensive to program. Large language models (LLMs) offer powerful reasoning capabilities, but are challenged by hallucinations, variable accuracy, and privacy concerns. We developed PromptNLP, an innovative hybrid approach using LLMs to author regex. This study compares PromptNLP’s performance in extracting apnea–hypopnea index (AHI) and Epworth Sleepiness Scale (ESS) versus manually coded regex, and validated its generalizability across institutions and note formats.

**Methods:** Sleep-related notes from KPSC, Beth Israel Deaconess Medical Center (BIDMC), and Emory University were used to extract AHI and ESS. PromptNLP involves two steps: (1) build an AHI/ESS phrase library (“Targeted” via manual effort, “General” via LLMs); (2) use the library to prompt an LLM to generate regex. This study was divided into three phases. Phase 1 compared multiple LLM-generated regex (ChatGPT-o3, Gemini-2.5Pro, Claude-Sonnet 4) against manually-developed regex. Phase 2 evaluated PromptNLP’s generalizability across institutions by applying the top-performing code from each site to a new institution and iteratively adapting it to the new note formats. Phase 3 applied the adapted regex on 207,404 KPSC notes and extractions were validated against 8,602 corresponding structured AHI.

**Results:** Phase 1: Targeted LLM regex outperformed manual regex in recall (AHI: 95–96% vs 80.8%; ESS: 98–100% vs 88%; P<0.05). The General approach also surpassed manual but with lower recall. Phase 2: Direct application to BIDMC performed poorly (AHI: 30%/ESS: 12%), but adapting the phrase library improved recall to 96%/84%. Applied to Emory, adapted regex recall was 85%/86%, increasing to 94%/89% after further adaptation. Phase 3: Among the 8,602 notes, AHI was extracted in 8,216 with 99.8% accuracy. Among the 207,404 notes, 105,823 had both AHI and ESS.

**Conclusion:** PromptNLP is an efficient, accurate, and generalizable LLM-based method for regex development in EHR phenotyping, outperforming manual approaches and enabling iterative improvement across health systems. This innovation could enable significant multicenter, large-scale sleep research by converting important but typically unstructured data into discrete metrics.

**006 — A buccal mucosal reflectance oximeter accurately measures arterial oxyhemoglobin saturation and pulse rate**

Presenter: Ming-Lai Lai, ProSomnus Sleep Technologies

Erin Mosca^1^, Connor Snow^2^, Matiram Pun^2^, Shane Magnison-Benoit^2^, Thomas Tripp^2^, Aimee Clarke^2^, Bradley Hansen^2^, Leo Transfiguracion^3^, Giovanni Di Simone^1^, Ming-Lai Lai^1^, Jean Rawling^2^, Steven Roy^2^, Saleema Adatia^4^, John Remmers^1^, Marc Poulin^2^

^1^ProSomnus Sleep Technologies, Pleasanton, CA; ^2^University of Calgary, Calgary, Canada; ^3^Alberta Health Services, Calgary, Canada; ^4^Symmetry Dental, Calgary, Canada

**Introduction:** Pulse oximeter devices (SpO_2_) suitable for multi-night monitoring of arterial oxyhemoglobin saturation (SaO_2_) are not readily available despite a need to monitor SaO_2_ decreases in patients with sleep apnea and pulmonary diseases during sleep. The objective of the current study was to assess the SpO_2_ and pulse rate accuracy of a buccal mucosal oximeter embedded into a custom-fitted overlay of the upper teeth by comparison to a gold standard (CO-oximeter SaO_2_ and 3-lead electrocardiogram [ECG] heart rate).

**Methods:** Twelve healthy participants (four females) aged 30.7±6.7 (22-39) years underwent a progressive stepwise desaturation protocol consisting of six 7-minute stages of decreasing levels of end-tidal oxygen (P_ET_O_2_; 90.1, 63.8, 53.0, 46.4, 41.6, and 37.9 Torr) to achieve a range of SaO_2_ from approximately 97-70% during semi-recumbent, non-motion conditions. P_ET_O_2_ and P_ET_CO_2_ were controlled by a dynamic end-tidal forcing function system. Arterial blood was drawn and analyzed using a Radiometer ABL800 CO-oximeter in the last three minutes of each stage. SpO_2_ and pulse rate values from the buccal mucosal oximeter were compared with CO-oximeter values of SaO_2_ and ECG heart rate, respectively.

**Results:** SaO_2_ values were evenly distributed over the range of 97-67%. Analysis of 325 CO-oximeter SaO_2_/buccal mucosal oximeter SpO_2_ data pairs yielded: r = 0.95; bias = 0.72; and accuracy root-mean-square (A_RMS_) = 2.94%. Analysis of 346 ECG heart rate/buccal mucosal oximeter pulse rate data pairs yielded: r = 0.99, bias = 0.30; and A = 2.08 bpm.

**Conclusions:** The buccal mucosal oximeter accurately measured SpO_2_ and pulse rate, as shown by good agreement with a gold standard, over a wide range of arterial hypoxemia. Such clinically acceptable accuracy indicates that this novel reflectance oximeter may prove useful in the management of patients with sleep-induced hypoxemia by providing multi-night monitoring of SaO_2_. Additionally, the intraoral placement of the oximeter might overcome some limitations of other pulse oximeters, as the buccal mucosa is highly vascularized, temperature regulated, protected from ambient light, and appears to lack melanin.

**Note**: Additional study results have been published in the journal Medical Devices (https://doi.org/10.2147/mder.s527510).

**007 — Screening for current major depressive episode (cMDE) using a single-lead II-ECG signal**

Presenters: Archie Defillo and Masimiliano Grassi, Medibio Limited

Archie Defillo^1^, Massimiliano Grassi^1^

^1^Medibio Limited

**Introduction:** Major depressive episodes (MDE) are prevalent, disabling, and often underdiagnosed, especially in patients with overlapping somatic or sleep-related complaints. Sleep disturbances and depression are closely linked, each exacerbating the other. Heart rate variability (HRV) derived from electrocardiogram (ECG) signals reflects autonomic dysfunction in depression and may serve as an objective biomarker. Advances in machine learning (ML) enable detailed analysis of ECG data, but its use in inpatient sleep medicine remains underexplored.

**Methods:** Lead II ECG signals (≥200 Hz) from PSG recordings, collected during Medibio’s Sleep Signal Analysis for Current Major Depressive Episode (SAMDE) Study (NCT05708222), were processed by a novel software prototype integrating two deep learning-based ECG sleep staging algorithms, HR/HRV metrics, and PHQ-9 items 1 and 2. These features were used to train an ML classifier for cMDE detection. Diagnostic accuracy was compared against the MINI and PHQ-9 ≥10 using sensitivity, specificity, predictive values, and McNemar’s test.

**Results:** The software showed 80.0% sensitivity (95% CI: 65.7–89.8), 71.0% specificity (95% CI: 64.6–76.3), 35.0% PPV, and 95.0% NPV relative to the Mini International Neuropsychometric Interview (MINI). PHQ-9 ≥10 yielded slightly higher sensitivity (87.8%) and NPV (96.6%), with comparable specificity (67.9%) and PPV (35.0%).

**Conclusion:** The ECG-based software demonstrates comparable accuracy to PHQ-9 for detecting cMDE, providing a promising, objective screening tool for sleep clinic populations.

**Poster Presentations**

**008 — Sleep and the skin**

Caris Talburt Fitzgerald, MD^1^

^1^University of Arkansas for Medical Sciences, Little Rock, AR

**Introduction:** Emerging research underscores a profound connection between sleep and skin health, reframing the skin not merely as a passive barrier but as a dynamic, sleep-sensitive organ. Mounting evidence suggests that sleep exerts widespread effects on cutaneous biology and may play a central role in both the pathogenesis and treatment of dermatologic disease.

**Methods:** This lecture synthesizes findings from over 70 peer-reviewed publications exploring the relationship between sleep and skin physiology. A comprehensive review of literature was conducted, drawing from dermatology, immunology, endocrinology, microbiology, and chronobiology sources. The review focused on the impact of sleep and circadian rhythms on inflammatory processes, immune function, microbiome diversity, hormonal pathways, genetic expression, and eccrine gland activity.

**Results:** Evidence indicates that poor sleep quality or disrupted circadian rhythms can drive systemic and cutaneous inflammation, impair skin barrier repair, and alter immune surveillance. Sleep loss has been shown to affect cortisol and melatonin levels, both of which play critical roles in skin homeostasis. Dysbiosis of the skin microbiome, changes in sebum and sweat production, and increased oxidative stress are all noted downstream consequences of inadequate sleep. These physiological shifts may contribute to the onset or exacerbation of a range of dermatologic conditions including acne, eczema, psoriasis, chronic wounds, autoimmune skin disease, and malignancy.

**Conclusion:** Sleep and circadian rhythms are integral to skin health, influencing a wide spectrum of dermatologic outcomes. Recognizing the skin as a sleep-responsive system introduces a novel axis for both prevention and treatment. A focus on sleep optimization offers translational promise in clinical dermatology, particularly within integrative and lifestyle medicine paradigms. This framework supports a future in which sleep assessment becomes a routine component of care across multiple disciplines including dermatology.

**009 — Likelihood of a current major depressive episode in individuals referred to sleep clinics for polysomnography (PSG) assessment using the MEB-001 algorithm**

Archie Defillo ^1^, Massimiliano Grassi ^1^

^1^Medibio Limited

**Introduction:** Depression and sleep disorders frequently co-occur, with sleep centers (SCs) documenting depressive symptoms in as many as 63% of patients and diagnosing depression in 22%. Despite this high prevalence, systematic depression screening is not routinely implemented in SCs. Medibio is developing technology designed to screen for current Major Depressive Episode (cMDE) in adults referred for PSG due to suspected obstructive sleep apnea (OSA), a population with elevated risk for depressive disorders.

**Methods:** MEB-001 was employed per Medibio’s Sleep Signal Analysis for Current Major Depressive Episode (SAMDE) Study (NCT05708222).

MEB-001 employs a series of locked machine learning algorithms, to analyze EEG and ECG signals to generate a cMDE screening output, which includes:

a) Meets Criteria for current MDE

b) Does Not Meet Criteria for current MDE

c) Unable to Assess current MDE

**Results:** MEB-001 achieved a sensitivity of 85.11% and specificity of 72.18% in detecting cMDE relative to the MINI. The positive predictive value was 37%, reflecting the relatively low prevalence of cMDE in the study cohort, while the negative predictive value was 96%, indicating strong capability to rule out cMDE in this setting.

**Conclusion:** MEB-001 algorithms offer the promise of a scalable, objective, and physiologically informed approach to depression screening in patients undergoing PSG for suspected sleep apnea. Its high sensitivity and negative predictive value support its potential role as a screening adjunct in sleep medicine, where timely identification of comorbid depression can facilitate earlier intervention and improve patient outcomes.

**010 — Effect of pharyngeal biostimulation on the upper airway in adults**

G. Dave Singh^1,2^, Shelby Wood^2^, Julienne Sabet^3^

^1^Division of Sleep Medicine, Stanford University, Stanford, CA; ^2^REMA Sleep Inc., Raleigh, NC; ^3^Developmental Dentistry, Chapel Hill, NC

**Introduction:** The mechanisms controlling pharyngeal airway behavior associated with obstructive sleep apnea (OSA) are poorly understood but flow limitation may play a role. The hypothesis tested in this pilot study is that biostimulatory changes induced in the pharyngeal complex may improve upper airway morphology.

**Methods:** Five volunteers (3 males and 2 females; aged between 46yrs and 65yrs) consented to participate in this study. All volunteers were scanned by a sonographer to gain echoarchitectural information of the end-tidal upper airway, using a hand-held ultrasound device (Clarius, Canada). Volunteers were subjected to initial biosignaling using a novel device (REMA Sleep, Inc.) for 15-20 mins. and immediately re-scanned. After daily use of the device for approx. 60 days, volunteers were re-scanned. From the ultrasound images, measurements were made in the lateral and axial planes to derive upper airway lengths, retroglossal cross-sectional areas (CSA) and upper airway volume measurements.

**Results:** The mean upper airway length increased from 6.15cm to 6.90cm in the region measured (from the skin surface to the base of the tongue), presumably indicating caudal traction, while the mean CSA increased from 17.4cm^2^ to 36.7cm^2^. Consequently, the mean expiratory upper airway volume increased from 88.8cm^3^ to 112.5cm^3^ after approx. 60 days of daily biostimulation.

**Conclusion:** Daytime pharyngeal biosignaling appears to induce beneficial changes in the upper airway in adults, which are similar to those reported using surgically-implanted ansa cervicalis stimulation procedures for OSA. Therefore, further investigations including overnight sleep studies are indicated and planned.

**Acknowledgments:** We would like to thank Jamila Battle for assisting in this work.

**011 — Comparative validation of consumer sleep trackers and polysomnography: a systematic review**

Elsie Osiogo^1^, Uwakmfonabasi Umoudoh^1^

^1^Lincoln Medical Center, Bronx, NY

**Introduction:** Widespread use of consumer wearable devices has made sleep data more accessible to all however, their accuracy and clinical usefulness in comparison to polysomnography (PSG) are still up for debate. Although PSG is still the gold standard, the need for home-based alternatives has increased due to its cost, intrusiveness, and impracticability for longitudinal monitoring. This systematic review synthesizes recent literature to critically evaluate the comparative accuracy and clinical utility of these devices.

**Methods:** We conducted a systematic review and retrospective analysis of studies validating wearable sleep trackers against PSG. Performance metrics evaluated included epoch-by-epoch agreement, Cohen’s Kappa, and sensitivity for sleep staging. In particular, we compared the effectiveness of devices that used direct electroencephalography (EEG) signals with those that used indirect signals (actigraphy, photoplethysmography [PPG]).

**Results:** Analysis of wrist-worn consumer devices demonstrated high sensitivity (≥95%) for identifying sleep versus wake. For gross parameters like total sleep time and sleep efficiency, these devices demonstrated good to excellent concordance with PSG. However, their accuracy for granular sleep staging was significantly lower, with sensitivities for deep and REM sleep as low as 50.5% in some studies. Device accuracy was also shown to decline in patient populations, particularly those with low sleep efficiency. In contrast, a review of a single-channel EEG-based wearable demonstrated substantial agreement with PSG for sleep staging (Kappa 0.69–0.79) and sensitivities above 79% for all sleep stages, suggesting that direct physiological measurement is superior.

**Conclusion:** In clinical decision-making, wearable trackers that rely on PPG and actigraphy are yet to act as a viable alternative to PSG. Although useful for monitoring general trends, their clinical utility is limited by their poor performance in patient populations and limited accuracy in sleep staging. We come to the conclusion that in order to achieve clinical-grade accuracy and genuinely turn home-based sleep monitoring into a revolutionary, useful tool for clinicians, future innovation should focus on devices with direct physiological sensing (such as EEG).

**012 — PROSOMNIA Sleep Therapy (PSTx™) anesthesia-induced REM sleep therapy: a neurological intervention for restorative sleep**

Nyree Penn^1^

^1^PROSOMNIA Sleep Health & Wellness, Aventura, FL

**Introduction**: Chronic insomnia and REM sleep deprivation remain among the most refractory disorders in clinical sleep medicine, contributing to cognitive impairment, mood dysregulation, and reduced quality of life. Current pharmacologic therapies act primarily at GABA-A receptors, producing sedation without reproducing the restorative neurobiology of REM sleep. In contrast, anesthesia engages both GABA-A and GABA-B receptors. This activation facilitates adenosine release, establishes REM-associated neurophysiology and utilizes glymphatic pathways for clearance. We present PROSOMNIA Sleep Therapy (PSTx™) — a disruptive, innovative intervention designed to intentionally induce restorative REM sleep in patients unresponsive to conventional therapies.

**Methods**: Adults with chronic insomnia and/or REM deprivation underwent monitored anesthesia care with a Propofol infusion. Infusion was titrated under continuous EEG monitoring (SedLine®) to achieve REM-specific waveform dominance. Sessions lasted 60–120 minutes under anesthesiologist supervision. Outcomes assessed pre- and post-therapy included validated sleep quality questionnaires, sleep onset latency, EEG-derived PSI variability, autonomic stability (HR, BP, respiratory variability), neurocognitive and mood measures (clarity, fatigue, mood scales), serum uric acid levels, and subsequent sleep continuity (patient-reported and wearable-derived).

**Results**: EEG data confirmed REM-state dominance with modulation of cortical hyperarousal. Patients demonstrated rapid neurologic recovery within minutes post-therapy. Neurocognitive benefits included sharper focus, improved clarity, and reduced fatigue. Sustained improvements were reported in subsequent nights, with enhanced sleep continuity and reduced reliance on pharmacologic therapies.

**Conclusion**: PSTx™ pioneers a new treatment category in sleep medicine: anesthesia-based sleep therapy. Unlike pharmacologic approaches that induce sedation, PSTx™ restores REM sleep architecture, re-establishes neurophysiologic homeostasis, and promotes glymphatic clearance of neurotoxic metabolites (adenosine, β-amyloid, tau). By bridging anesthesiology and sleep medicine, PSTx™ reframes REM sleep itself as a precision therapeutic modality for treatment-resistant insomnia- advancing the frontier of restorative sleep interventions.

**Support:** PROSOMNIA Sleep Health & Wellness

**013 — Use of a high-fidelity physiologic data platform as a screening modality for sleep-disordered breathing in a hospitalized patient with congenital heart disease**

Andrew Ligsay, MD^1,2^, Marco Almeda, MD^2^

^1^Division of Pediatric Cardiology, Ann & Robert H. Lurie Children’s Hospital, Chicago, IL; ^2^Division of Pediatric Pulmonary & Sleep Medicine, Ann & Robert H. Lurie Children’s Hospital, Chicago, IL

**Introduction:** Sleep-disordered breathing is associated with increased morbidity in hospitalized patients with congenital heart disease (CHD). As such, timely identification of sleep-disordered breathing may help improve clinical outcomes in this population. We describe a case of a 6-year-old with a history of hypoplastic left heart syndrome and serial palliative surgical interventions who ultimately underwent orthotopic heart transplant. The patient was found to have severe obstructive sleep apnea (OSA) one month status-post transplant after concern for sleep-disordered breathing was identified through use of a continuous high-fidelity physiologic data recording platform.

**Methods:** A retrospective analysis was performed of prospectively collected physiological data from the above patient who was admitted to the cardiac intensive care unit at Ann & Robert H. Lurie Children’s Hospital (Chicago, IL). Biomarkers, including pulse oximetry, heart rate, and heart rate variability, were collected and stored using Sickbay software (Medical Informatics Corp). This information was also compared to simultaneous polysomnography data collected by Easy III software (Cadwell Industries, Inc). Respiratory events were identified on polysomnography data per American Academy of Sleep Medicine pediatric scoring criteria for sleep-disordered breathing.

**Results:** Initial review of the patient’s Sickbay data revealed a baseline nocturnal oxygen saturation of 96% with periodic clusters of brief oxygen desaturation events occurring at 60-to-120-minute intervals. Oxygen saturations largely decreased to the low 90s% with some saturations decreasing to the mid 80s%. A polysomnography study was subsequently performed for this patient, which revealed severe obstructive sleep apnea (oAHI = 19.7/hour) which was worse during REM stage sleep (REM AHI = 69.2/hour). Baseline oxygen saturation during the polysomnography study was 95% with an oxygen saturation nadir of 79%. Review of simultaneous Sickbay data during the polysomnography study similarly identified brief periods of oxygen desaturation that matched scored respiratory events on the polysomnography analysis.

**Conclusion:** Continuous high-fidelity physiologic data recording software can be a useful screening tool to identify cases of sleep-disordered breathing in hospitalized patients with congenital heart disease. Additional studies are recommended comparing sensitivity and specificity of this screening modality to polysomnography analysis.

**014 — Tonic motor activation (TOMAC) as a platform for restless legs syndrome (RLS) treatment: evaluating opportunities to increase potency and duration**

Hussein Alawieh^1^, Kurtis Swartz^1^, Leticia Camacho^1^, John Colborn^1^, Stephanie Rigot^1^, Jonathan Charlesworth^1^

^1^Noctrix Health

**Introduction:** Restless legs syndrome (RLS) is a neurological disorder that disrupts sleep and quality of life. A new category of wearable neuromodulation technology – tonic motor activation (TOMAC) – has been shown to reduce RLS symptoms and improve sleep quality by stimulating the peroneal nerve. Only one device in this category has been tested – the Nidra**®** TOMAC system. Here, we evaluate if alternative forms of TOMAC generated by the same neuromodulation platform could result in greater potency or duration of RLS relief.

**Methods:** Nidra**®** delivers bilateral high-frequency electrical stimulation to the peroneal nerve for 30-minutes at intensities at or below 40mA. It is powered by a rechargeable battery that typically allows for two 30-minute sessions per charge. Here, we tested if intensities above 40mA (HI-TOMAC) could increase acute relief of RLS symptoms. We also tested if modulated stimulation waveforms (M-TOMAC) could increase the number of sessions per charge. We enrolled 17 adults with moderate-severe RLS with prior exposure to ≥12 weeks of the Nidra**®** system in a prior study; stronger responders from this prior study (n=9) were assigned to 2-wks of M-TOMAC; weaker responders (n=8) were assigned to 2-wks of HI-TOMAC. The likelihood of significant acute relief was defined as the percentage of sessions with participant global impression of improvement (PGI-I) ratings of much improved or very much improved.

**Results:** HI-TOMAC increased the likelihood of significant acute relief by 43.7% (95% CI: 27.1% to 60.3%, p<0.001). M-TOMAC increased number of sessions per charge by a factor of 12.2x (95% CI: 10.1 to 14.1, p<.001) with no reduction in the likelihood of significant acute relief (95% CI: -25.7% to +28.5%, p=.91). There were no serious device-related adverse events and no dropouts due to tolerability or adverse events.

**Conclusions:** These results suggest that there may be opportunities to extend the TOMAC neuromodulation platform to provide greater acute relief of RLS symptoms and >10x longer duration of therapy per charge. Further research would be helpful to understand which subgroups of patients could benefit from these investigational features.

**Support:** This study was funded by Noctrix Health, Inc.

**Note**: Additional study results have been published in the Journal of Clinical Medicine (https://doi.org/10.3390/jcm15082845).

**015 — A reflectance oximeter embedded in a dental overlay can accurately quantify the efficacy of mandibular advancement device therapy**

Len Liptak^1^, Ming-Lai Lai^1^, Giovanni Di Simone^1^, Wendy Weisshaar^1^, Christoper Holland^1^, Saleema Adatia^2^, Erin Mosca^1^

^1^ProSomnus Sleep Technologies, Pleasanton, CA; ^2^Symmetry Dental, Calgary, Canada

**Introduction**: Pulse oximeter devices suitable for multi-night monitoring of conditions such as obstructive sleep apnea (OSA) are not readily available. ProSomnus Sleep Technologies (Pleasanton, CA) previously developed a buccal mucosal pulse oximeter embedded into a custom-fitted overlay of the upper teeth. The objective of the current pilot study was to assess the accuracy of the buccal mucosal oximeter in individuals with OSA to determine if the device produces an accurate oxygen desaturation index (ODI) when used in conjunction with a mandibular advancement device.

**Methods**: Eight volunteers with OSA wore a buccal mucosal and reference (WristOx 3150, Nonin Medical Inc., Plymouth, MN) oximeter overnight in the home for two nights. The upper dental overlay of the buccal mucosal oximeter was worn on the first night to simulate untreated OSA (no mandibular protrusion) and a lower mandibular advancement device arch was added for the second night to provide mandibular protrusion in the range of 30-70% (partial or full treatment).

**Results**: Sixteen nights of data were collected. Linear regression showed good concordance between the buccal mucosal and reference oximeters (r = 0.94; p < .000001). The median ODI was not significantly different between the buccal mucosal (28.3 events/h) and reference oximeter (32.4 events/h) with the upper dental overlay (p = 0.2656) or when both the upper dental overlay and a lower mandibular advancement device arch were used (buccal mucosal oximeter: 12.6 events/h; reference oximeter: 13.7 events/h; p = 0.4609). The difference between the untreated and treated position was significant for both oximeters (p < 0.05 for both). The OSA severity category matched in 81.3% of cases.

**Conclusions**: The results of this pilot study indicate that a reflectance oximeter embedded in a dental overlay can report an accurate ODI in individuals with OSA both when used on its own and when used in conjunction with a mandibular advancement device, demonstrating that this technology has the potential to objectively quantify the efficacy of mandibular advancement device therapy. Further research is needed to validate these results in a larger population against a gold standard.

**016 — Obstructive sleep apnea and panic disorder: a critical review of the bidirectional comorbidity**

Katherine Shiells^1^

^1^Case Western Reserve University, Cleveland, OH

**Introduction:** Obstructive sleep apnea (OSA) has a population prevalence of 2-4%, and is a sleep disorder that includes repetitive interruptions in breathing during sleep. Symptoms include sudden awakening, increased sympathetic arousal, sleep fragmentation, and daytime sleepiness, and are connected to the future development of significant health disorders. Panic Disorder (PD) is an anxiety disorder that includes symptoms of intense fear, increased heart rate, elevated blood pressure, and decreased quality of life. 18% of panic attacks occur from a sleeping state, and so, the comorbidity of obstructive sleep apnea and panic disorder is extremely significant in effective clinical treatment of patients. Panic attacks and OSA events can also occur in similar sleep stages (non-REM) with shared symptoms of shortness of breath and sudden awakenings. This paper aims to examine the bidirectional relationship between obstructive sleep apnea and panic disorder in the population and analyze the current, yet limited, literature on the subject.

**Methods:** Twelve empirical studies were selected for this review, using the keywords: Sleep apnea or obstructive sleep apnea or apnea; panic disorder or panic or nocturnal anxiety.

**Results:** The studies determined that individuals with both OSA and PD presented with increased respiratory irregularities, which worsened individuals’ sleep quality, arousal levels, somatic symptoms, and anxiety. CPAP therapy was shown to significantly reduce symptoms in both OSA and PD, especially nocturnal panic, somatic arousal, and insomnia, increasing sleep quality and daytime functioning.

**Conclusion:** Future longitudinal studies should aim to increase sample sizes, as diagnostic and treatment technology is now allowing a deeper analysis of the comorbidities that these two conditions are associated with. Increased awareness of OSA-PD comorbidity is essential for timely and accurate diagnosis. Increased awareness in research and clinical practice of the bidirectional relationship between OSA and PD is essential to emphasize how both conditions exacerbate each other’s symptoms through shared physiological, neurological, and psychological mechanisms. An integrated clinical approach that considers both sleep and mental health may offer significant improvements in patient outcomes and quality of life.

**017 — Utilization of the Papillary Bleeding Index (PBI) by dental professionals as a rapid screening tool for obstructive sleep apnea (OSA)**

Katherine C. Schacherl^1,2^

^1^Marquette University School of Dentistry, Milwaukee, WI; ^2^University of Oxford, United Kingdom

**Introduction:** The current estimate of 7,500 sleep physicians at a provider to patient ratio of 1:43,000 (Watson et al., 2017) is not a realistic solution to managing the considerable burden of OSA in the United States (US). Beginning in 2017, the >200,000 active, licensed US dentists were “encouraged” by the American Dental Association (ADA) to screen patients for sleep-related breathing disorders (SRBD) (ADA 2017, 2024). Current laws forbid dentists from diagnosing patients with OSA.

The SleepImage Ring is an FDA-cleared home sleep test to aid health care professionals in diagnosis and management of sleep-disordered breathing (SleepImage 501(k) clearance 2019). It may be used by dentists within their scope of practice for screening patients for SRBD.

A repeatable, easy-to-implement workflow for dental professionals to screen and refer patients for suspected OSA would decrease the burden on healthcare services.

**Methods:** Bleeding gums are neither normal nor healthy. In the absence of local irritants (e.g. plaque or calculus) they are even more concerning. Research by Schacherl et al. (2024) found the Papillary Bleeding Index (PBI) was a more reliable OSA dental screening tool than STOP-Bang or Epworth Sleepiness Scale (ESS) questionnaires. In this novel workflow the PBI is used by dental professionals as a rapid clinical screening tool for OSA. If the screening results in suspicion of OSA, the patient will complete validated questionnaires (e.g. STOP-Bang, ESS) and the SleepImage Ring dispensed for sleep screening. A referral will be made to a sleep physician who will review the cloud-based sleep data along with the patient’s dental and medical records to provide a diagnosis.

**Results**: The PBI is able to correctly identify >60% of dental patients with OSA compared with STOP-Bang (<19%) and ESS (<7%) which makes it a powerful screening tool (Schacherl et al., 2024).

**Conclusion:** Utilization of a novel dental workflow to screen patients for OSA will decrease the burden on healthcare services in the US and expediate patient care.

**018 — Novel therapeutic oral device for sleep apnea**

Phinarak Hao^1^

^1^13 Little Ladies Inc., Jacksonville, FL

**Introduction:** FreePap treats OSA and improves snoring by preventing the collapse of the soft palate and tongue in the upper airway, which is the most common cause of OSA. FreePAP is a revolutionary oral appliance. It has the stability and anchoring benefits of a mandibular advancement device but does not pull on the teeth or lower jaw. And it functions by stabilizing the tongue similar to tongue-retaining/stabilizing devices without protruding the tongue outside the mouth. It supports the upper airway dilator muscles to maintain airway patency. The airway channel is an H beam design that supports a space between the tongue and soft palate and posterior pharyngeal wall. The device is custom fit to each patient's individual dentition and oropharynx.

The oral appliance displaces the base of the tongue from the posterior pharyngeal wall and breaks contact between the tongue and palate. The device helps retain the tongue in an anterior position and maintains airway patency between the tongue and soft palate and encourages patency of the nasopharynx along with the oropharynx.

The H beam design of the airway channel is mated to the entire surface of the palate. This results in pressure from the tongue being distributed over the widest surface area possible on the tongue and palate, and minimizing uncomfortable pressure points anywhere in the mouth. The oral appliance is custom fitted to the entire upper dentition and palatal vault in order to maximize comfort and overnight retention of the device. It is made entirely of the softest medical grade silicone possible for comfort.

**Methods:** An intraoral 3D scan of the teeth and palate, in combination with cone beam CT are used to design a completely customized device for each patient. These devices are printed by advanced 3D silicone printers.

**Results:** Preliminary cone beam CT scans of prototypes confirm airway patency and are very promising. Once patients are acclimated, the device becomes very comfortable.

**Conclusion:** We need a better alternative therapy to surgical hypoglossal nerve stimulators, CPAP, and mandibular advancement devices which have very poor compliance.

**019 — Expanding access to cognitive behavioral therapy for insomnia through a virtual, PSYPACT-authorized practice model**

Erin K. Baehr^1^

^1^Insight Sleep Specialists, Cockeysville, MD

**Introduction:** Insomnia represents a major public health challenge as a sleep disorder impacting up to 30 percent of the population, with strong links to depression, anxiety, cardiovascular disease, and reduced quality of life. Cognitive Behavioral Therapy for Insomnia (CBT-I) is the gold-standard treatment, yet access is limited by geography, provider shortages, and systemic barriers. Innovative delivery models are needed to expand access to this highly effective treatment.

**Methods:** Insight Sleep Specialists, LLC is a fully virtual insomnia clinic directed by a PhD clinical psychologist, double board certified in both Sleep Medicine and Behavioral Sleep Medicine. As a PSYPACT-authorized provider with an Authority to Practice Interjurisdictional Telepsychology (APIT) and an ASPPB E.Passport certificate, the clinic has the ability to provide CBT-I services across more than 40 participating states and territories.

The model integrates: Telepsychology to overcome geographic barriers. Standardized sleep tracking and structured assessment tools to guide treatment. Data-driven and customized CBT-I protocols for efficient, personalized care. Scalable infrastructure for integration with health systems, sleep centers, and employer wellness programs.

**Results:** In less than 5 years, this model has expanded access to care, reaching patients across 14 different states who may otherwise not have access to an equal quality and specialty of care. Engagement and completion rates are high because the model eliminates barriers such as commuting, rigid clinic hours, and limited local options, while offering flexibility for visits before work or outside traditional office hours. This accessibility supports sustained participation, with clinical improvements consistent with CBT-I benchmarks, including reduced sleep onset latency, decreased wake after sleep onset, increased total sleep time, improved subjective sleep quality, and lower rates of sleep anxiety. Patients also report reduced hypnotic medication use and improved daytime functioning. Importantly, this model has extended access to patients in underserved areas, reaching individuals who lack local behavioral sleep medicine providers.

**Conclusion:** A PSYPACT-enabled, virtual insomnia clinic demonstrates an innovative care model that transforms access to behavioral sleep medicine. By leveraging APIT authority, digital tools, and a scalable delivery framework, this model has the potential to expand high-quality insomnia care nationally and improve population sleep health.

**020 — SOMNAR: a vibroacoustic anatomic polysomnogram for full-night mapping of upper-airway collapse in OSA**

Onur Kilic^1^, Meagan R. Pitcher^1^, Britt Cross^1^

^1^Bairitone Health, Houston, TX

**Introduction** Targeted therapies for obstructive sleep apnea succeed only when matched to anatomy and physiology during natural sleep. PSG/HST quantify severity but not collapse sites; drug-induced sleep endoscopy (DISE) is invasive and diverges from real sleep. We developed SOMNAR, a low-profile, skin-mounted array that noninvasively produces an anatomic polysomnogram, a synchronized, night-long map of multilevel airway behavior aligned to sleep stage, body position, respiratory effort, and airflow. By adding an anatomical layer to routine PSG/HST, SOMNAR shifts testing from event counts to mechanism-aware phenotyping that can guide therapy.

**Methods**: Three synchronized contact accelerometers on a facial patch record tissue-borne vibrations during sleep. Pairwise relative transfer functions capture craniofacial propagation, yielding signatures independent of vibration or snore source. Wake speech anchors calibrate the representation and normalize inter-subject variability. A self-supervised contrastive encoder learns latent airway states, which are mapped with a small DISE-labeled set to anatomically interpretable collapse categories defined by VOTE (velum, oropharyngeal lateral walls, tongue base, epiglottis), including degree and configuration. Validation used leave-one-subject-out cross-validation in a DISE cohort (N=20) with per-patient majority-vote summaries. Usability was assessed in two pilot home studies with oximetry and position.

**Results**: Per-patient summary outputs agreed 82% with DISE, comparable to 88% surgeon-surgeon agreement. Presence of obstruction: kappa 0.46 at the palate and 0.49 at the hypopharynx. Degree 0/1/2: linear-weighted kappa 0.70 at the palate and 0.48 at the hypopharynx. SOMNAR captured dynamic changes during DISE consistent with waning propofol effect, e.g., a shift from concentric to anteroposterior velar collapse several minutes after induction, concordant with the surgeon. In home testing, application required less than 10 minutes, recordings synchronized to timelines and desaturation events, and there were no device-related adverse events.

**Conclusion**: SOMNAR delivers a noninvasive, interpretable, full-night, context-tagged map of upper-airway collapse, i.e., an anatomic polysomnogram, during natural sleep. By coupling anatomy to physiologic context, it can distinguish primary from secondary collapse and has potential to improve patient selection and titration for targeted therapies such as upper airway stimulation, oral appliances, positional therapy, and surgery.

**Support**: This work was supported by NIH Grant R43HL168994-01. FDA pre-submission feedback informed study design.

**021 —** The LunAIRo trial: a 51-week phase 3, randomized, double-blind, placebo-controlled study of aroxybutynin and atomoxetine (AD109) in obstructive sleep apnea

Sanjay R. Patel^1^, Ron Farkas^2^, Luigi Taranto-Montemurro^2^, John Cronin^2^, Patrick J. Strollo Jr^1,3^

^1^ Division of Pulmonary, Allergy, Critical Care and Sleep Medicine, Department of Medicine, University of Pittsburgh, Pittsburgh, PA; ^2^Apnimed Inc, Cambridge, MA; ^3^ Veteran Affairs Pittsburgh Healthcare System, Pittsburgh, PA

**Introduction:** Neuromuscular dysfunction contributes to the pathophysiology of obstructive sleep apnea (OSA) driving recurrent upper airway collapse during sleep. A Phase 3 trial of AD109, an investigational, oral neuromuscular modulator combining the antimuscarinic, aroxybutynin (2.5 mg), with the selective norepinephrine reuptake inhibitor, atomoxetine (75 mg), evaluated the efficacy and safety in OSA.

**Methods:** LunAIRo (NCT0811247) was a randomized, double-blind, placebo (PBO)-controlled, 51-week, parallel-arm Phase 3 trial of AD109 vs PBO in adults with mild-to-severe OSA intolerant to or refusing positive airway pressure therapy. Key inclusion criteria included apnea-hypopnea index ≥5 (AHI_4_), and BMI 18–40 kg/m^2^ (males)/ 18–42 kg/m^2^ (females). Participants were randomized 1:1 to AD109 or PBO. Efficacy assessments were analyzed in intention-to-treat (ITT; participants receiving ≥1 dose of treatment) and modified intention-to-treat sets (mITT; ITT participants having ≥1 post-baseline polysomnogram on treatment) at Weeks 26 (primary) and 51 (secondary). AD109 safety and tolerability was evaluated through Week 51.

**Results:** Participants (N=660) were randomized (AD109: n=329; PBO: n=331) with 524 (80.0%) completing study. In the mITT (AD109: n=240; PBO: n=293), AD109 significantly reduced AHI_4_ (LS mean [95% CI]) at Week 26 by −5.8 (−7.5, −4.2) vs −0.0 (−1.6, 1.5) with PBO (p<0.0001), representing improvements of 46.8% at Week 26 (vs 6.8%, PBO; p<0.001) and 43.5% at Week 51 (vs 13.2%, PBO; p<0.001). In the ITT (AD109: n=324; PBO: n=326), AD109 significantly reduced AHI_4_ by −3.9 (−5.4, −2.4) vs 0.2 (−1.2, 1.7) for PBO (p<0.001). In the mITT, AD109 significantly improved select secondary endpoints at Week 26, including change in oxygen desaturation index – ODI_3_ (LS mean [95% CI]; −7.5 [−9.4, −5.7] vs −0.1 [−1.7, 1.6]; events/hr; p<0.0001) and relative change in hypoxic burden – HB_4_ (58.2% vs 13.8%; p<0.0001). AD109 significantly reduced AHI_4_ ≥50% at Week 26 in 38.3% of participants (vs 15.4%, PBO; p<0.0001) in the mITT. Adverse events (AEs) were predominantly mild, with ≥1 AE reported by 272 (82.9%) participants with AD109 and 223 (68.2%) with PBO. No treatment-related serious AEs were identified.

**Conclusion:** LunAIRo demonstrates AD109’s therapeutic benefit for participants with mild-to-severe OSA across multiple weight classes.

**Support:** Funded by Apnimed, Inc.

**022 —** The SynAIRgy trial: a 26-week phase 3, randomized, double-blind, placebo-controlled study of aroxybutynin and atomoxetine (AD109) in obstructive sleep apnea

Patrick J. Strollo Jr^1,2^, Ron Farkas^3^, Luigi Taranto-Montemurro^3^, John Cronin^3^, Sanjay R. Patel^2^

^1^ Veteran Affairs Pittsburgh Healthcare System, Pittsburgh, PA; ^2^ Division of Pulmonary, Allergy, Critical Care and Sleep Medicine, Department of Medicine, University of Pittsburgh, Pittsburgh, PA; ^3^Apnimed Inc, Cambridge, MA

**Introduction:** Obstructive sleep apnea (OSA) is characterized by neuromuscular dysfunction causing recurrent upper airway collapse during sleep. A Phase 3 trial of AD109, an investigational, oral neuromuscular modulator combining the antimuscarinic, aroxybutynin (2.5 mg), with the selective norepinephrine reuptake inhibitor, atomoxetine (75 mg), assessed the efficacy and safety in participants with OSA.

**Methods:** SynAIRgy (NCT05813275) was a randomized, double-blind, placebo (PBO)-controlled, 26-week, parallel-arm Phase 3 trial of AD109 vs PBO in adults with mild-to-severe OSA intolerant to or refusing positive airway pressure therapy. Key inclusion criteria were apnea-hypopnea index **≥**5 (AHI_4_; 4% hypopnea desaturation) and BMI 18–40 kg/m^2^ (males) / 18–42 kg/m^2^ (females). Participants were randomized (1:1) to AD109 or PBO. Efficacy endpoints were analyzed in the intention-to-treat (ITT; all randomized participants receiving ≥1 dose of treatment) and modified intention-to-treat sets (mITT; participants in ITT having ≥1 post-baseline polysomnography on treatment). Safety and tolerability of AD109 was evaluated.

**Results:** Participants (N=639) were randomized (AD109: n=319; PBO: n=320) with 539 (84.6%) completing the trial. In the mITT (AD109: n=260; PBO: n=296), AD109 significantly reduced AHI_4_ (LS mean [95% CI]) at Week 26 by −6.1 (−7.8, −4.4) vs 0.4 (−1.2, 2.0) with PBO (p<0.0001), representing a 55.6% improvement (vs 19.1%, PBO; p<0.0001). In the ITT (AD109: n=302; PBO: n=313), AD109 significantly reduced AHI_4_ by −3.3 (−5.1, −1.5) vs 0.7 (−1.0, 2.4) for PBO (p=0.001). In the mITT, AD109 significantly improved select key secondary efficacy endpoints at Week 26, including change in oxygen desaturation index - ODI_3_ (LS mean [95% CI]; −6.5 [−8.4, −4.6] vs 0.7 [−1.1, 5.5] events/hr; p<0.0001) and relative change in hypoxic burden - HB_4_ (60.5% vs 14.7%; p<0.0001). AD109 significantly reduced AHI_4_ by ≥50% at Week 26 in 39.6% of participants (vs 22.3%, PBO; p<0.0001) in the mITT.

Adverse events (AEs) were predominantly mild with ≥1 AE reported by 232 (71.4%) participants with AD109 and 151 (47.3%) with PBO. No treatment-related serious AEs were identified.

**Conclusion:** SynAIRgy demonstrates that AD109 provides therapeutic benefit for participants with mild-to-severe OSA across a range of weight classes.

**Support**: Funded by Apnimed, Inc.

**Note**: Additional study results have been published in the American Journal of Respiratory and Critical Care Medicine (https://doi.org/10.1093/ajrccm/aamag215).

**023 — Rationale and design of a phase 3 open-label extension study of fixed dose combination of aroxybutynin and atomoxetine (AD109) in obstructive sleep apnea**

Patrick J. Strollo Jr^1^, Sanjay R. Patel^2^, Stephen Pittman^3^, Ketan Mehta^3^, John Cronin^3^, Luigi Taranto Montemurro^3^, Ronald Farkas^3^

^1^Department of Medicine, University of Pittsburgh Medical Center, Pittsburgh, PA; ^2^Division of Pulmonary, Allergy, Critical Care and Sleep Medicine, University of Pittsburgh, Pittsburgh, PA**;** ^3^Apnimed Inc., Cambridge, MA

**Introduction:** Obstructive sleep apnea (OSA) is characterized by sleep-related neuromuscular dysfunction and predisposing anatomic abnormalities that result in hypoxia due to repetitive collapse of the pharyngeal airway. AD109 is an investigational, first-in-class, oral neuromuscular modulator that combines the antimuscarinic, aroxybutynin (2.5 mg), and the selective norepinephrine reuptake inhibitor, atomoxetine (75mg) designed to mitigate airway collapse. Two randomized, double-blind, placebo-controlled phase 3 trials, the 26-week SynAIRgy (NCT05813275) and 51-week LunAIRo (NCT05811247), evaluated the efficacy, safety, and tolerability of AD109 vs placebo in participants with mild-to-severe OSA. We have followed up with an open-label extension (OLE) study (NCT06566820) to evaluate the long-term safety of AD109. In addition, we aimed to assess night-to-night OSA variability with a high-resolution pulse oximetry ring and the OSA burden over time using validated Patient Reported Outcomes (PROs) collected with a mobile application.

**Methods**: All participants completing a parent phase 3 trial with continued use of investigational product were eligible if considered safe by the study team. Safety assessments include continued measurement of vital signs, monitoring and recording of adverse events, serious adverse events, pregnancies, and recording of study or treatment discontinuations. Optional exploratory data collection involving serial assessments using a ring-worn pulse oximeter and OSA-related PROs were offered to the OLE participants. Oximetry-derived parameters included the oxygen desaturation index based on 4% desaturation, time with oxygen saturation ≤ 90%, and hypoxic burden.

**Results:** Six hundred and three participants (approximately 70% of eligible) have enrolled in the OLE study to date. Four hundred and sixty-four participants (77%) also opted-in to the exploratory data collection with 28,847 overnight pulse oximetry recordings and 4,635 PRO questionnaires collected.

**Conclusion:** This large OLE study is designed to confirm and extend the safety evidence of the phase 3 SynAIRgy and LunAIRo randomized control trials evaluating the efficacy and safety of AD109 in the treatment of OSA. We have observed substantial participation and adoption for multiple-night use of digital tools including pulse oximetry and PROs to characterize OSA burdens, both objective and subjective.

**Support:** This study is funded by Apnimed, Inc.

**024 — Efficacy of virtual-only visits for OSA in longitudinal CPAP compliance**

Subaila Zia^1^

^1^Telemedora

**Introduction:** Obstructive sleep apnea (OSA) affects millions of Americans. Yet timely access to sleep care including diagnosis and treatment remains a challenge leading to significant co-morbidity and economic burden. Our study explores virtual only care as the pathway for sleep care. We hypothesize that virtual care is effective and non-inferior to managing patients with OSA and could be used as a reliable care model for longitudinal care.

**Methods:** A retrospective chart review of adult patients with OSA for whom CPAP was prescribed between March 2022 to September 2024 was done. Data collected included baseline demographics, results of home sleep apnea testing/polysomnogram, Epworth sleepiness score, CPAP compliance and residual AHI. Patients were seen only virtually and by the same board-certified sleep physician. Inclusion criteria: a. Adults (≥18 years) virtually seen for suspected OSA or those for management of previously diagnosed OSA. b. Patients who were treated with CPAP/BiPAP therapy. c. Patients who had been seen for at least 12 months and had 12-month CPAP compliance data available. We excluded patients who were prescribed CPAP but did not return for follow-up at 1 year, those who had change of modality from CPAP to BiPAP.

**Results:** Of a total of 21 patients, 12 (57%) were males and 9 (43%) were female, median age of 40.5 years and median BMI of 30.55. Patients had mild to severe OSA. The percentage of patients who met CMS CPAP compliance criteria, i.e. >4 hours of CPAP use at least 70% of nights at 12 months was approximately 66%. All patients had residual AHI of less than 5 except 1. These results are at par or somewhat better than what has been reported nationally for in-person care at 1 year. Some studies reported 1 year CPAP compliance at 50-55%. However, our study showed 12-month CPAP compliance of 66%.

**Conclusion:** With shortage of board-certified sleep physicians, virtual care delivery as sole form of care delivery for OSA as treatment option and for longitudinal care.

**025 — The WhisperSom™ PST-1™ for sleep apnea: one device to test, treat, and track**

Audrey Wells¹, Craig F. Feied^1^, David B. Goldstein^1^, Michael G. Nathans^1^

¹WhisperSom Medical Technologies, Annapolis, MD

**Introduction:** The WhisperSom™ PST-1™ device represents a disruptive innovation across the continuum of sleep apnea care: multi-night diagnosis, adaptive treatment, and ongoing monitoring with real-time responsiveness during sleep. PST-1 (currently v8) is a wearable multisensor and auditory neuromodulation device with no components on the face or in the mouth, nose, or neck. In diagnostic mode, it measures breathing patterns and deviations to determine sleep apnea severity and hypoxic burden. In therapy mode, algorithmic early detection of apneas/hypopneas triggers precisely timed acoustic signals delivered to subcortical respiratory networks. These stimuli act on hypoglossal and phrenic nerve pathways, increasing airway tone and diaphragmatic excursion to terminate events before significant desaturation and sympathetic activation. PST-1 continuously measures events and biomarkers, enabling self-auditing algorithms to adapt therapy within milliseconds in a closed-loop system.

**Methods:** Feasibility studies evaluated efficacy, tolerability, and safety in 7 volunteers with mixed obstructive and central sleep apnea over 125 in-home test nights, recording physiologic responses to 2,470 recorded apneas: 1,281 un-treated / 1,189 treated with the PST-1.

**Results:** Technical and clinical success was demonstrated. PST-1 reduced apneas >10 seconds by 93% and >20 seconds by 99%. Oximetry showed a 97% reduction in sleep time below 90%. Although not the primary outcome, AHI decreased by 24%. Motion and heart rate increases were reduced, and patients reported high comfort and acceptance compared to PAP therapy, with no compromise in sleep quality. Multi-night recording improved diagnostic yield and enabled longitudinal tracking of treatment response.

**Conclusion:** PST-1 redefines sleep apnea management: a single device that can diagnose, treat, and monitor all forms of sleep apnea. By integrating precision acoustic neuromodulation with real-time physiologic feedback, PST-1 can expand access, improve adherence, and substantially reduce the burden of untreated sleep apnea on patients, providers, and healthcare systems.

**Support:** Supported by WhisperSom internal research and development.

**Figure:**
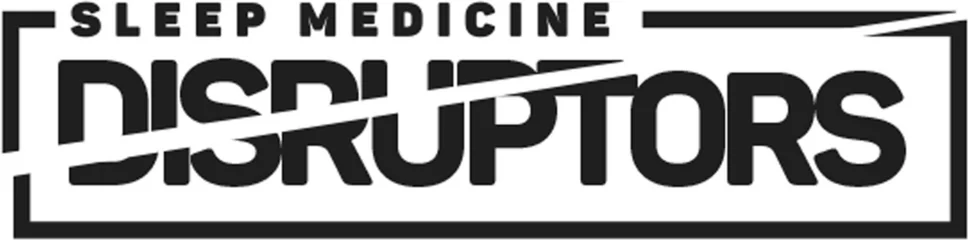


**Figure:**